# A Rare Case of Spontaneous Healing of an Anterior Cruciate Ligament Tear

**DOI:** 10.7759/cureus.80171

**Published:** 2025-03-06

**Authors:** Daniel R Baka, Erastus Thuo, Veniamin Barshay, Dug Su Yun

**Affiliations:** 1 Orthopaedics, Cooper University Hospital, Camden, USA; 2 Radiology, Cooper University Hospital, Camden, USA; 3 Sports Medicine, Cooper University Hospital, Camden, USA

**Keywords:** acl injuries, acl tear, anterior cruciate ligament (acl), conservative therapy, spontaneous healing

## Abstract

Anterior cruciate ligament (ACL) injuries are among the most common knee injuries in the United States. The ACL is an intra-articular ligament that resists anterior tibial translation and provides rotational stability. Most ACL injuries occur through non-contact mechanisms and are typically diagnosed based on history, physical examination, and confirmatory MRI. Treatment options include operative and non-operative management, with the latter focusing on restoring functional stability rather than expecting the ACL to heal spontaneously.

In this case report, we present a 33-year-old female patient with a sedentary lifestyle who experienced a popping sensation and immediate swelling after twisting her knee while sitting down. She presented to the clinic one week later, reporting knee instability and a dull, aching pain rated 7/10. Physical examination revealed pain with passive knee extension and positive patellar compression, Clarke’s inhibition, McMurray’s, and Lachman’s tests. MRI confirmed a complete ACL tear and a full-thickness cartilage defect in the medial facet of the patella. The patient opted for non-operative treatment, including a crossover knee brace and physical therapy. Over multiple follow-up visits, her range of motion and pain improved. Eleven months post-injury, a follow-up MRI ordered to evaluate a suspected reinjury unexpectedly revealed a completely intact ACL, indicating spontaneous healing.

This case highlights a rare instance of spontaneous ACL healing in a sedentary adult who chose conservative management. Although non-operative therapy typically aims to restore function rather than facilitate ligament healing, emerging evidence suggests spontaneous ACL healing is possible, particularly in proximal femoral single-bundle tears. Further research is needed to establish standardized conservative treatment protocols that optimize outcomes and promote ACL regeneration.

## Introduction

Anterior cruciate ligament (ACL) injuries are among the most common knee injuries, with more than 200,000 cases occurring annually in the United States [1]. The ACL is an intra-articular ligament in the knee composed of two distinct bundles: the anteromedial and posterolateral bundles [2,3]. Its primary function is to restrain anterior translation of the tibia while also contributing to rotational stability [2,3]. Anatomically, the ACL originates from the medial intercondylar notch of the lateral femoral condyle and attaches to the tibial plateau [2,3]. It receives its blood supply primarily from the middle genicular artery, with additional contributions from the inferomedial and inferolateral genicular arteries, and it is predominantly composed of Type I collagen [2].

More than 40% of ACL injuries occur in non-contact scenarios [1]. High-risk movements include cutting and planting, sudden changes in direction or speed, landing from a jump, twisting, and pivoting motions [4]. Clinical diagnosis typically relies on a detailed history and physical examination, with confirmation provided by magnetic resonance imaging (MRI) [4]. Common physical exam tests used to assess ACL integrity include the anterior drawer test, Lachman test, and pivot shift test [2,4].

Management of ACL injuries may be surgical or non-surgical, with treatment decisions based on patient demographics, injury severity, and long-term functional goals [5]. Despite extensive research, there is no definitive consensus on the superiority of one approach over the other, and treatment should be individualized to optimize patient outcomes. With conservative therapy, the primary goal is to restore functional stability rather than facilitate ligament healing [6]. Spontaneous ACL healing is not typically expected and there have been no reported cases of spontaneous healing in non-athletic patients [6].

In this case report, we present the evaluation and management of a patient with a confirmed ACL tear on MRI who opted for conservative treatment. Remarkably, follow-up imaging 11 months later revealed an intact ACL, suggesting spontaneous healing.

## Case presentation

A 33-year-old female presented to our clinic one week after sustaining a non-traumatic right knee injury. The injury occurred while she was sitting and twisted her knee, hearing a popping sound followed by swelling. She sought treatment at the emergency department (ED) the same day, where she was placed in a knee immobilizer. At her initial clinic visit, she reported knee instability and episodes of the knee giving way. She described her pain as a dull ache, rated 7/10, which worsened with prolonged walking but improved with rest and analgesics.

**Figure 1:**
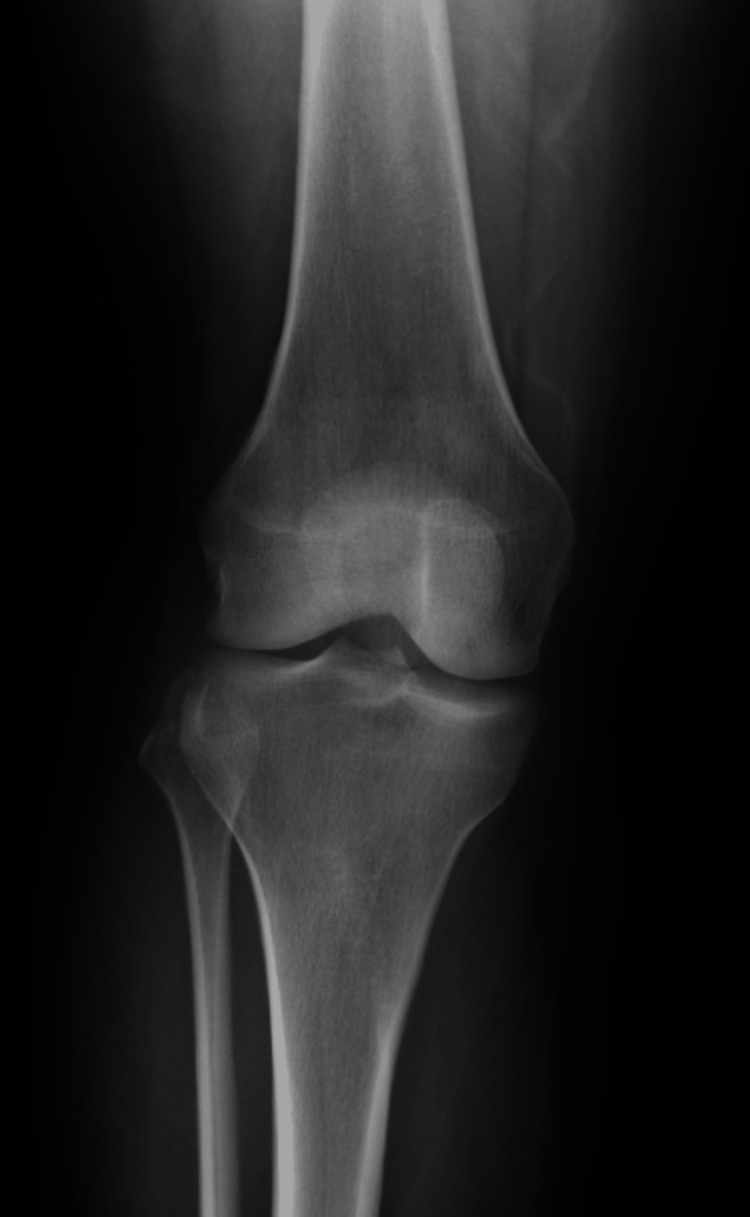
AP radiograph of the patient’s right knee a few hours after injury AP: Anteroposterior

**Figure 2:**
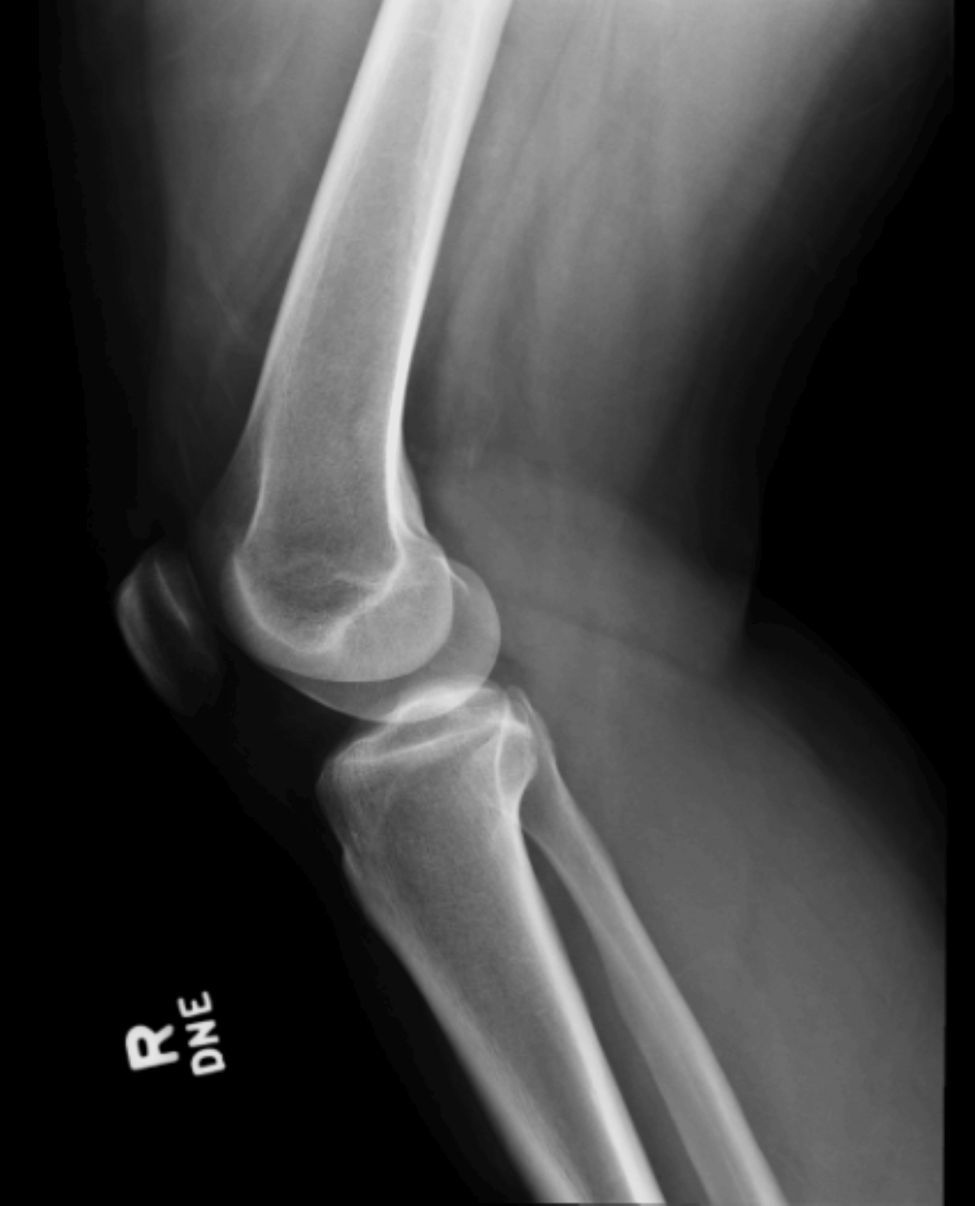
Lateral radiograph of the patient’s right knee a few hours after injury

**Figure 3:**
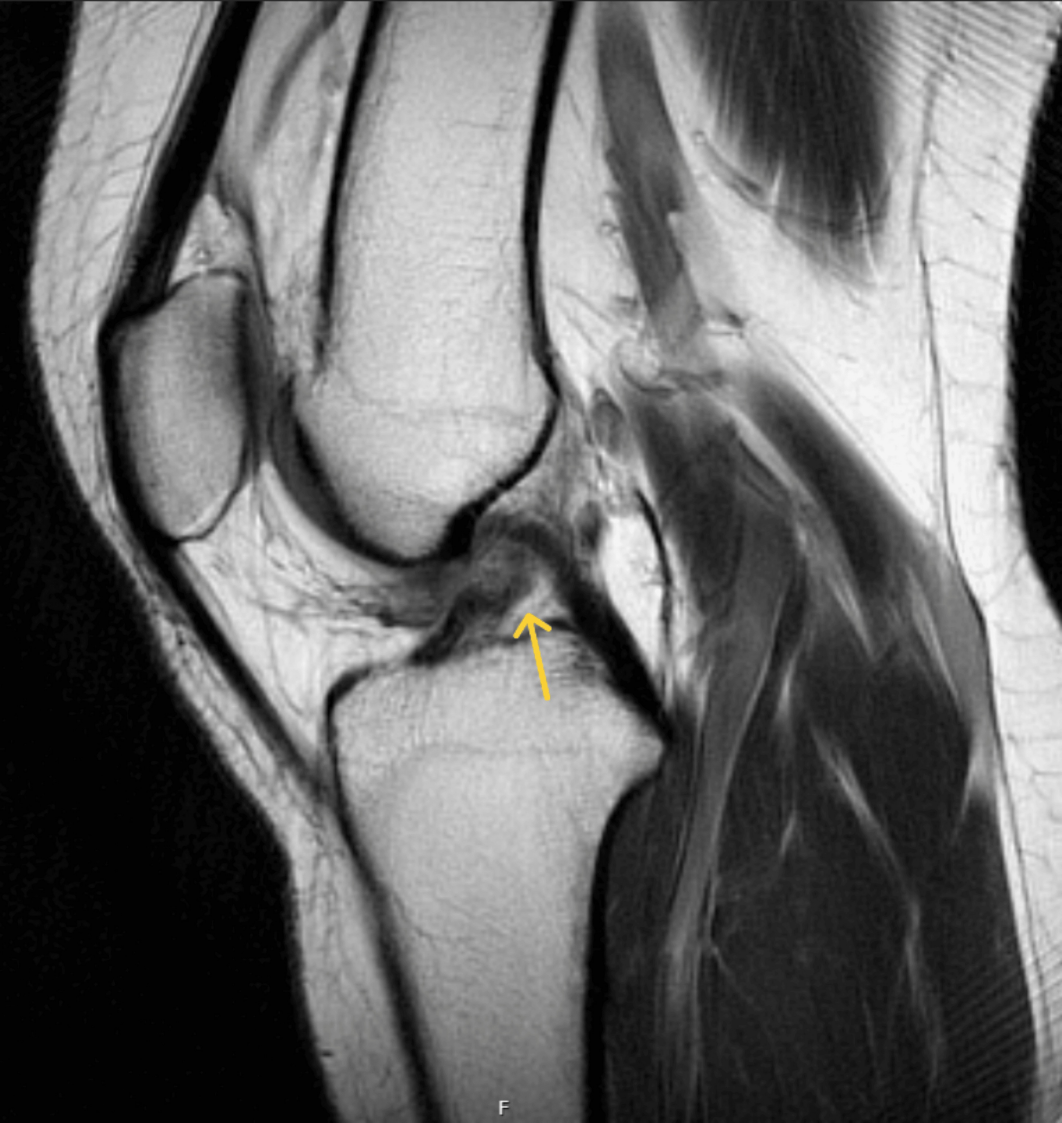
Sagittal proton density MRI of the right knee demonstrating an ACL tear (yellow arrow) ACL: anterior cruciate ligament

**Figure 4:**
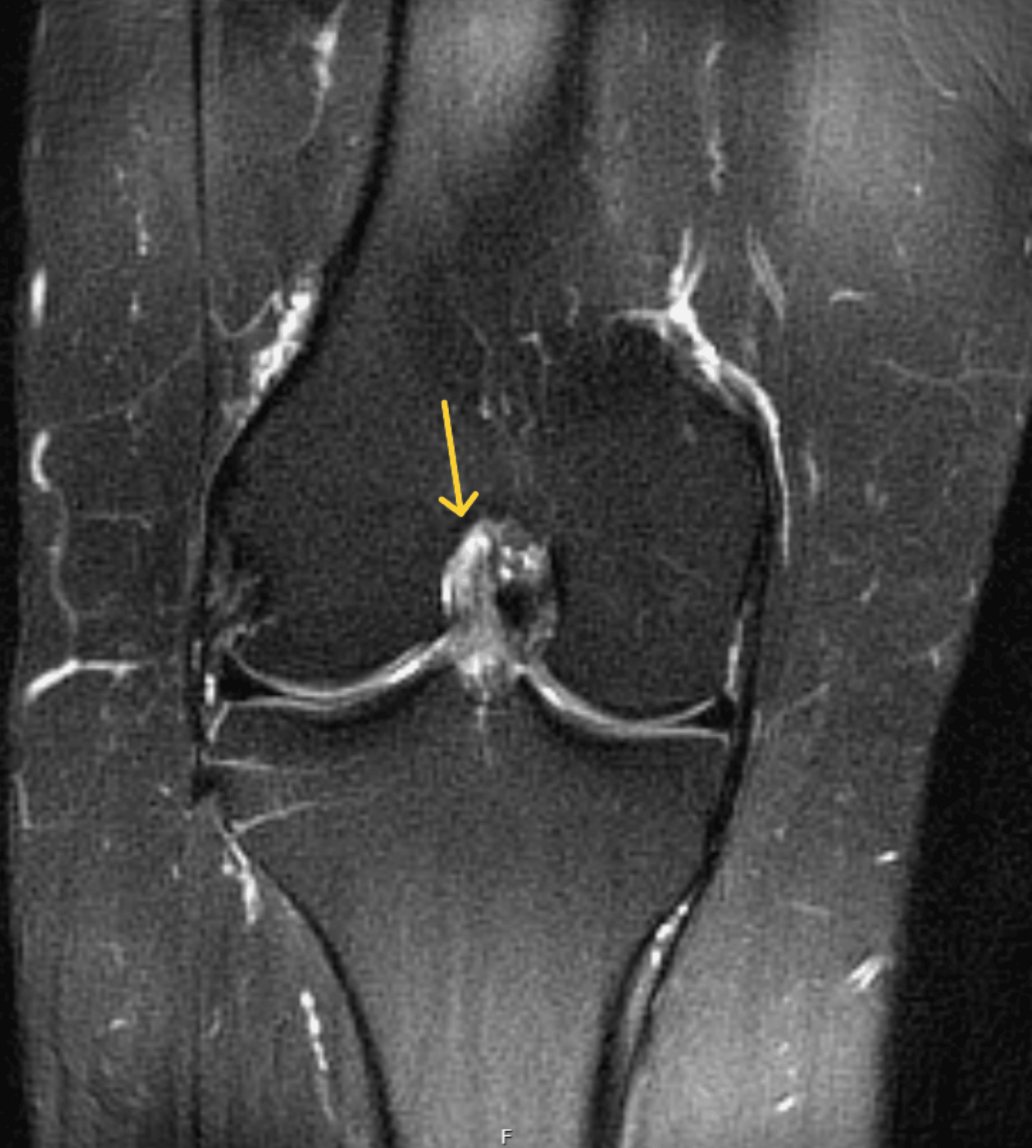
Coronal T2-weighted fat-saturated MRI of the right knee demonstrating an ACL tear (yellow arrow) ACL: anterior cruciate ligament

**Figure 5:**
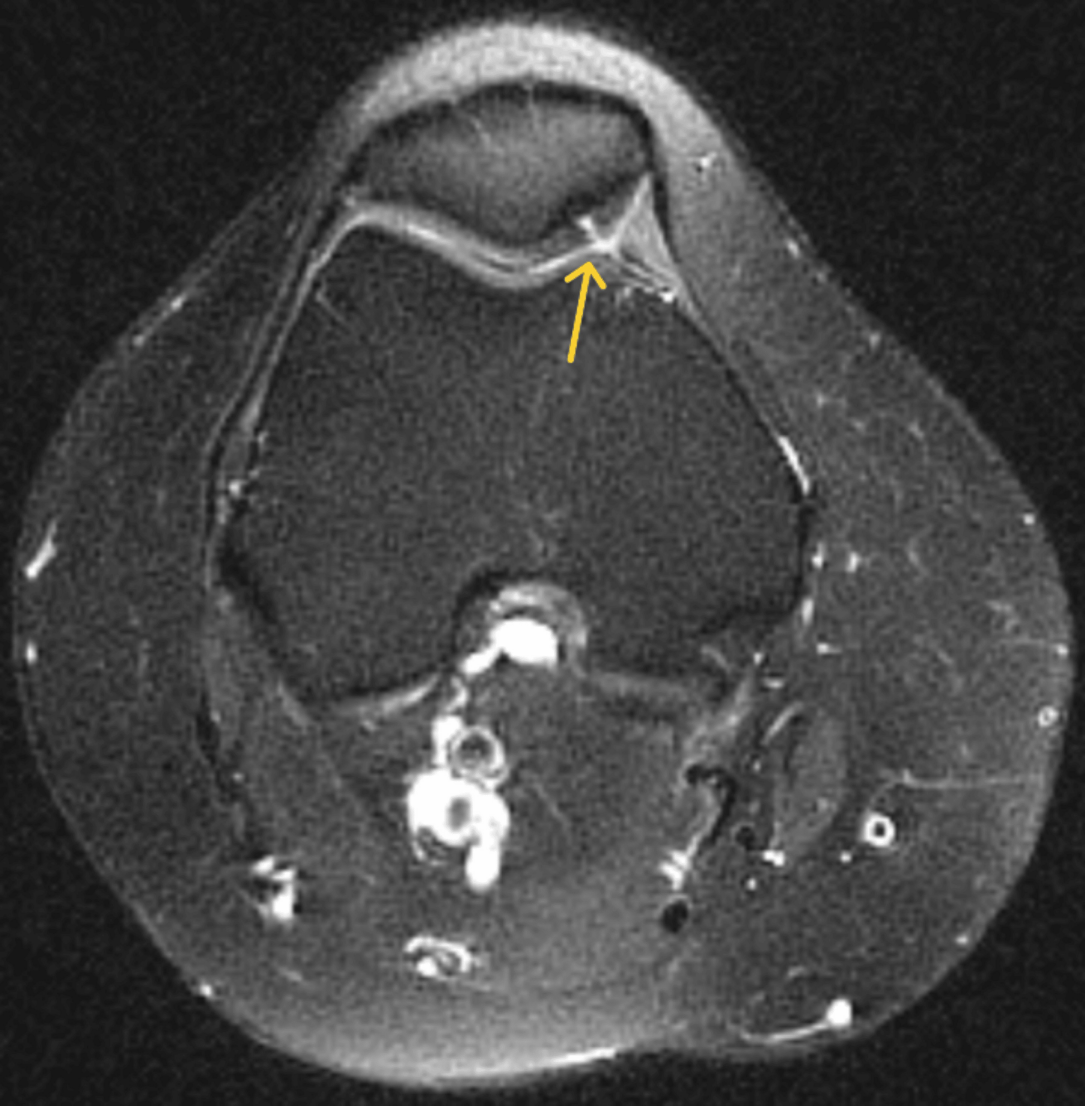
Axial T2 of the right knee showing a full-thickness fissure in the medial patellar cartilage (yellow arrow)

On physical examination, there was mild effusion (+1) and tenderness over both the medial and lateral joint lines. Her right knee range of motion (ROM) was 0-100° with pain on passive extension. Special tests were positive for patella compression, Clarke’s inhibition, McMurray’s, and Lachman’s tests. X-rays obtained in the ED showed no fractures or dislocations (Figures 1, 2). An MRI performed 10 days post-injury confirmed a complete ACL tear along with a full-thickness defect in the articular cartilage of the medial facet of the patella (Figures 3-5).

Treatment options, including surgical and non-surgical management, were discussed with the patient. Given her sedentary lifestyle, she opted for non-operative treatment but requested a consultation with a surgeon for further evaluation. This consultation occurred one month post-injury, after which she decided to continue with conservative management. She was referred for physical therapy, placed on work restrictions, and fitted with a crossover hinged knee brace.

At her six-week follow-up, after two weeks of physical therapy, she reported no knee pain. Examination revealed resolution of knee effusion and joint line tenderness. ROM improved to 0-120° without pain on passive extension. Patella compression, Clarke’s inhibition, and Lachman’s tests remained positive, while McMurray’s test was negative. She was cleared to return to work and continue physical therapy.

At her two-month follow-up, she had completed physical therapy and reported minimal pain, rating it 2/10. Examination findings were unremarkable, with ROM unchanged. Patella compression, Clarke’s inhibition, and Lachman’s tests remained positive. She was advised to continue home exercises and follow up as needed.

At 7.5 months post-injury, she experienced a sudden popping sensation during knee extension, followed by increased pain. She returned to the clinic at eight months post-injury with pain rated 5/10. Examination revealed medial joint line tenderness but no significant changes in ROM. Patella compression and Clarke’s inhibition tests remained positive, while Lachman’s test was negative. A cortisone injection was administered, and a crossover knee brace was provided. She was advised to follow up in one month.

At her nine-month follow-up, she continued to report knee pain, still rated 5/10. Examination showed tenderness along both the medial and lateral joint lines, but ROM remained stable. Patella compression, Clarke’s inhibition, McMurray’s, and Lachman’s tests were positive. Given her worsening symptoms and the prior popping sensation, there was concern for a possible meniscus injury, prompting an MRI.

**Figure 6:**
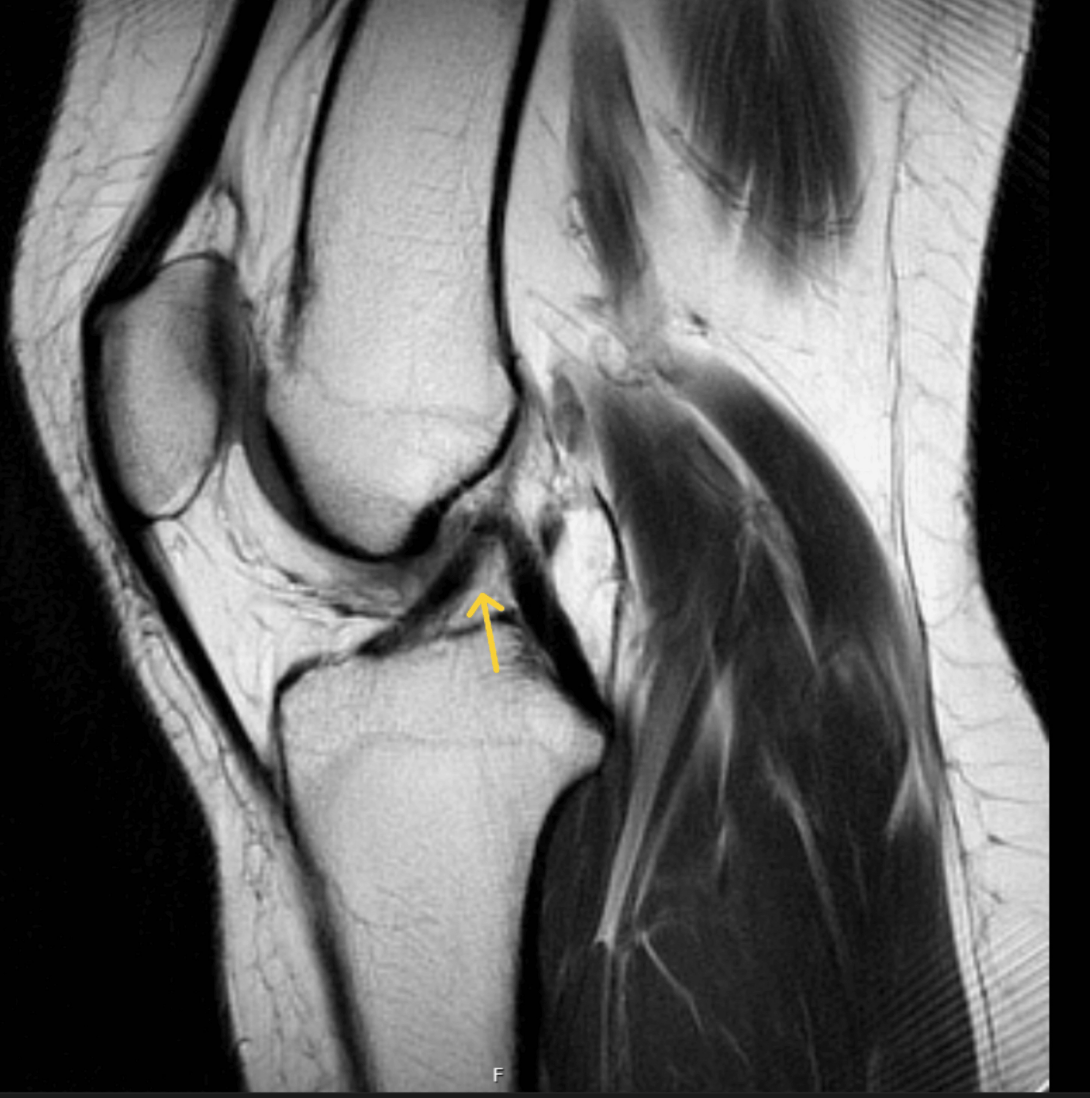
Sagittal proton density MRI of the right knee demonstrating an intact ACL (yellow arrow) ACL: anterior cruciate ligament

**Figure 7:**
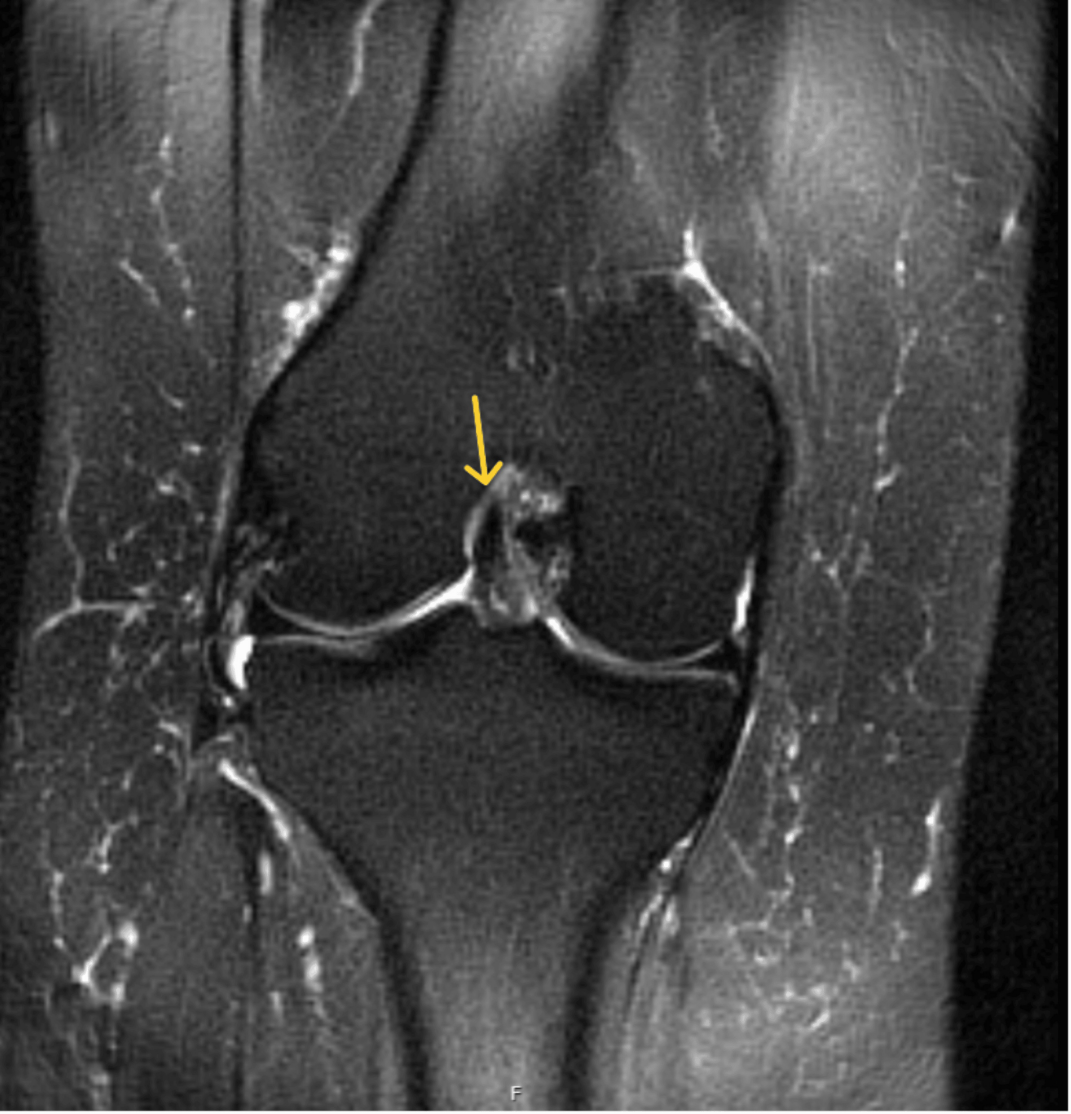
Coronal T2-weighted MRI of the right knee demonstrating an intact ACL (yellow arrow) ACL: anterior cruciate ligament

**Figure 8:**
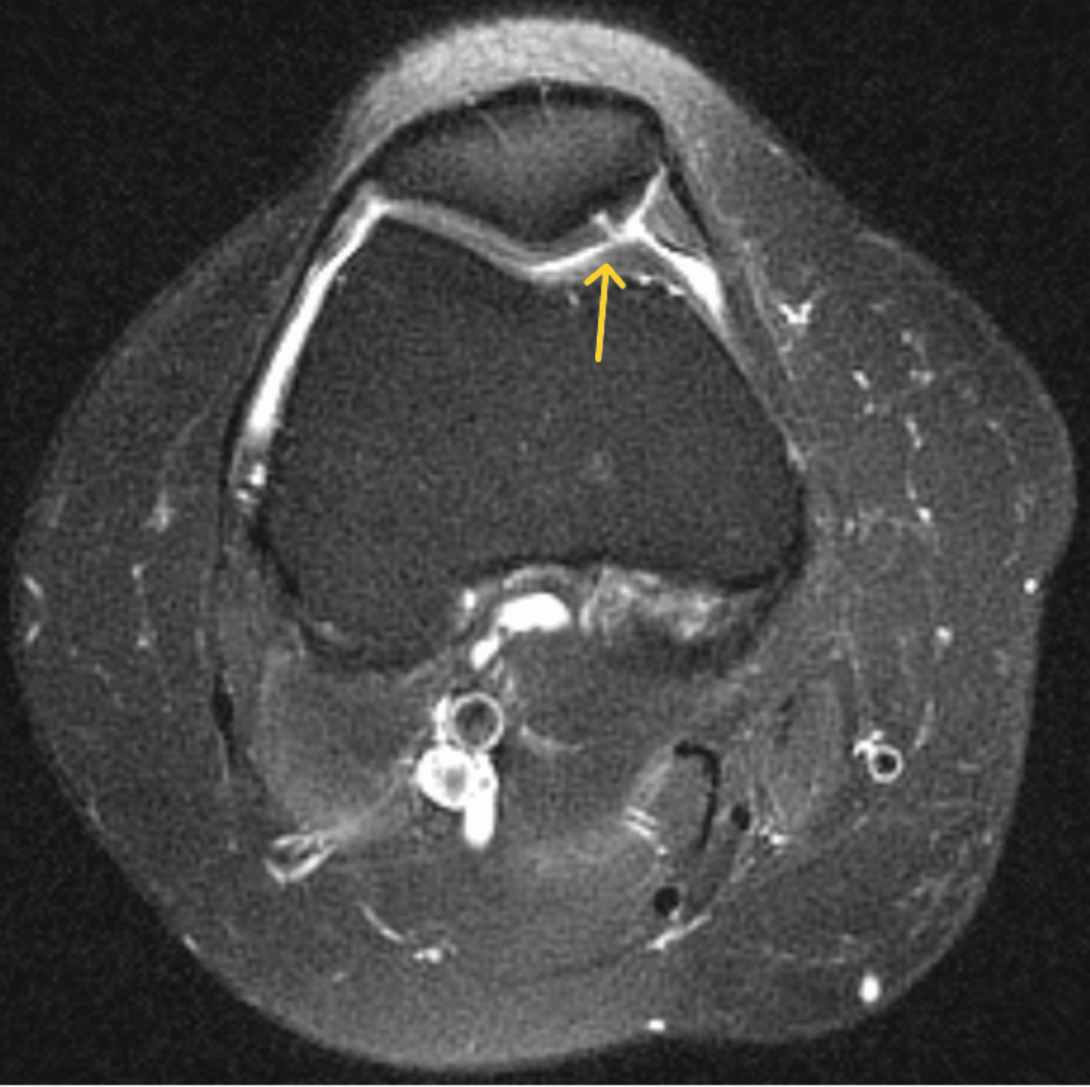
Axial T2-weighted MRI showing a full-thickness fissure in the medial patellar cartilage (yellow arrow)

At her 11-month follow-up, she continued to experience knee pain, rated 5/10. Examination findings remained largely unchanged, with no knee instability, medial and lateral joint line tenderness, and stable ROM (0-120°). Patella compression and Clarke’s inhibition tests were still positive, while Lachman’s test remained negative. MRI, performed 10.4 months post-injury, unexpectedly revealed decreased signal intensity compared to before, and an intact ACL with evidence of a healed tear, as well as a full-thickness fissure in the articular cartilage of the medial patellar facet (Figures 6-8). She declined further cortisone injections but was referred for continued physical therapy and prescribed diclofenac gel.

## Discussion

We present a case of a 33-year-old female who experienced a "pop" and immediate knee swelling after twisting her knee while attempting to sit on a public bus. ACL tears should be strongly suspected in patients who report hearing a “pop” at the time of injury, particularly when accompanied by immediate swelling, pain, and knee instability. Physical examination plays a critical role in diagnosis, with tests such as the anterior drawer, Lachman, and lever tests being commonly used. While earlier studies reported 100% sensitivity and specificity for the lever test, a more recent blinded cross-sectional study found the Lachman and anterior drawer tests to be superior in specificity, with the Lachman test also demonstrating greater sensitivity [7,8]. In this case, the patient had a positive Lachman test, raising high clinical suspicion, which was later confirmed on MRI as a full-thickness ACL tear.

Following an ACL tear diagnosis, treatment decisions should involve shared decision-making, considering injury severity, patient activity level, concomitant injuries, and long-term goals [9]. As noted in the introduction, studies report minimal differences in outcomes between conservative therapy and surgical reconstruction of the ACL. A meta-analysis found that while surgery resulted in less knee laxity, it was also associated with a higher incidence of osteoarthritis, with no significant differences in patient-reported outcomes [10].

The KANON study, which followed 121 young, active adults with acute ACL tears, compared structured rehabilitation with early ACL reconstruction to structured rehabilitation with optional delayed surgery. Of the latter group, 36 patients did not undergo surgery; however, after two years, there was no significant difference in Knee Injury and Osteoarthritis Outcome Scores (KOOS) between the groups [11].

In this case, the patient had an isolated ACL tear and a sedentary lifestyle, making conservative therapy an appropriate choice. When opting for non-operative management, the goal is to restore knee stability, as the ACL itself is not expected to reattach [6]. Histological studies by Murray et al. suggest that the ACL has poor healing capacity due to a lack of bridging tissue between torn segments. After an ACL tear, inflammatory cells migrate to the injury site, followed by fibroblast proliferation and synovial sheath formation around the distal ACL stump; however, reattachment typically does not occur [12].

Despite histological evidence suggesting poor healing capacity, Blanke et al. analyzed 381 ACL tears and found that 14% healed spontaneously. Their findings suggest that femoral single-bundle lesions and minimal posterior tibial slope increase the likelihood of healing [13]. Notably, our patient had a femoral single-bundle lesion with a posterior tibial slope of 0-1°, aligning with these favorable parameters.

Other reports have described spontaneous ACL healing, though most are based on small case series, and no reports have been published on spontaneous ACL healing in a middle-aged, sedentary female. Previ et al. studied six recreational athletes with proximal third ACL tears treated with a knee extension brace for three weeks, followed by at least two months of physiotherapy. MRIs at six months showed end-to-end ACL continuity in all cases (Howell grades I and II) [14]. Costa-Paz et al. retrospectively examined 14 athletically active patients with proximal third and mid-ligament complete ACL ruptures who underwent unspecified physical therapy without bracing. At the final follow-up, all had an intact ACL and were able to return to sports, though two patients experienced retears [15]. Our patient was instructed to use a cross-over brace and attend physical therapy which could have aided in spontaneous ACL healing. However, there are no direct guidelines on how to treat ACL tears conservatively to maximize healing, so it is uncertain the influence therapy and bracing had on the healing potential. 

Historically, the ACL was considered incapable of healing; however, these reports challenge that notion and suggest the potential for spontaneous recovery in select cases [13-15]. Unfortunately, there are no established guidelines for conservative treatment to optimize healing. Further research is needed to explore the role of bracing, physiotherapy protocols, and other factors in promoting ACL regeneration.

## Conclusions

This case highlights a rare instance of spontaneous ACL healing in a sedentary 33-year-old female who opted for conservative management. After almost a year of conservative treatment, the patient reported that she was back to her functional status before the injury and was not experiencing any knee instability; however, she still had pain in her knee. While ACL tears can be treated surgically or non-surgically, conservative therapy traditionally focuses on restoring function and stability rather than ligament healing. However, emerging evidence suggests that spontaneous healing may occur, particularly in cases of single-bundle proximal femoral tears. Additionally, patient activity level, biological variability, physical therapy, and bracing may all influence healing potential. Currently, no standardized conservative treatment protocols exist to optimize healing. Further research is needed to establish effective non-operative strategies and develop guidelines to promote spontaneous ACL regeneration.
